# Selective outcome reporting across psychopharmacotherapy randomized controlled trials

**DOI:** 10.1002/mpr.1900

**Published:** 2021-11-11

**Authors:** Michelle Lancee, Marleen Schuring, Joeri K. Tijdink, An‐Wen Chan, Christiaan H. Vinkers, Jurjen J. Luykx

**Affiliations:** ^1^ Department of Psychiatry UMC Utrecht Brain Center University Medical Center Utrecht (UMCU) Utrecht University Utrecht The Netherlands; ^2^ Department of Philosophy Vrije Universiteit Amsterdam Amsterdam The Netherlands; ^3^ Department of Medicine Women's College Research Institute University of Toronto Toronto Ontario Canada; ^4^ Department of Psychiatry Amsterdam UMC Vrije Universiteit Amsterdam Amsterdam Public Health research institute and Amsterdam Neuroscience Amsterdam The Netherlands; ^5^ Second Opinion Outpatient Clinic GGnet Mental Health Warnsveld The Netherlands; ^6^ Department of Psychiatry and Neuropsychology School for Mental Health and Neuroscience, Maastricht University Medical Centre Maastricht The Netherlands

**Keywords:** outcome reporting bias, psychiatry, research ethics, selective outcome reporting

## Abstract

**Objective:**

Selective reporting impairs the valid interpretation of trials and leads to bias with regards to the clinical evidence. We aimed to examine factors associated with selective reporting in psychopharmacotherapy trials and thus enable solutions to prevent such selective reporting in the future.

**Methods:**

We retrieved all registry records of trials investigating medication for depressive, bipolar and psychotic disorders. Multivariate logistic regression was performed with selective reporting as outcome, and funding source, psychiatric disorder, year of study start date, participating centers, and anticipated sample size as explanatory variables, after testing for multicollinearity. Adjusted odds ratios (AOR) were calculated. Two‐sided Fisher exact test was used to compare the proportions of newly added positive primary outcomes with the proportions of positive results in the overall group of primary outcomes.

**Results:**

Of 151 included trials (*N* = 94,303 participants), 21 (14%) showed irregularities between registered and published primary outcomes. Higher odds of such irregularities were associated with non‐industry‐funded RCTs (AOR 5.3; *p* = 0.014) and trials investigating major depressive disorder (AOR 12.7; *p* = 0.024) or schizophrenia (AOR 14.5; *p* = 0.016; Table 1).

**Conclusion:**

We demonstrate discrepancies between trial registrations and publications across RCTs investigating debilitating psychiatric disorders, especially in non‐industry funded RCTs.

## 1 INTRODUCTION

In 2000, the National Institutes of Health (NIH) created the registry and results database Clinicaltrials.gov. The FDA Amendments Act of 2007 mandates registration and submission of randomized controlled trials (RCTs) to Clinicaltrials.gov (Zarin et al., 2015). One aim of RCT registration is to minimize selective reporting of clinical trial outcomes (Chan et al., 2017; Roest et al., 2015; Turner, 2013). Selective reporting impairs the valid interpretation of trials and leads to bias with regards to the clinical evidence (Ioannidis et al., 2017). In the field of psychiatry, selective reporting was demonstrated by our group for antipsychotics in schizophrenia and schizoaffective disorders (Lancee et al., 2017) and by others for antidepressants in anxiety disorders (Roest et al., 2015). However, a systematic analysis of selective reporting in trials of schizophrenia spectrum, bipolar and depressive disorders is currently lacking. These specific disorders were chosen as they represent a large group of well‐defined psychiatric disorders with specific pharmacotherapy treatments consisting of antipsychotics, antidepressants and mood stabilizers. Our primary objective was to investigate factors associated with selective outcome reporting in the selected disorders. Our secondary objective was to examine whether selective outcome reporting is associated with the directionality of primary outcomes. We performed systematic analyses targeting primary and secondary outcomes to ultimately provide a starting point for possible solutions.

## 2 METHODS

We performed a search on 31 August 2019 on Clinicaltrials.gov using the key words “schizophrenia OR schizoaffective disorder AND antipsychotics”, “antidepressant or mood stabilizer and depressive disorder or bipolar disorder”. Studies registered from 1 January 2006 to 31 December 2015 were included. This registration date was chosen in light of the mandated WHO registration of clinical trials since 2006. Inclusion criteria for registry records were: study phase II–IV; completed randomized controlled trials; trials including either major depressive disorder, bipolar disorder, schizophrenia or schizoaffective disorder; and FDA‐approved medication given as investigational compound for that diagnosis. Both prospectively and retrospectively registered records were included. Trials were excluded when registry records had no matching publication on Pubmed, Embase or CIHNAL before 31 August 2019 (retrieved through NCT number), or when they did not meet inclusion criteria (i.e., other study designs, interventions or diagnoses).

All publications matching the NCT number were retrieved. If more than one publication for a certain registry record was found, we included the publication that in our opinion most accurately represented the purpose of the study mentioned on Clinicaltrials.gov. Data extraction procedures were according to our previous publication (Lancee et al., 2017).

Primary and secondary outcomes were compared between registry records and publications. Outcomes were considered primary outcome measures when they were stated as such in the publication, or when they were the main focus of the article corresponding with the clearly defined primary outcome measures on Clinicaltrials.gov. We extracted the variables listed in Table 1 for all retrieved publications from clearly defined sections on Clinicaltrials.gov. Selective outcome reporting was defined by two separate authors as adding, changing or deleting of an outcome measure. Discrepancies were resolved by discussions with the other co‐authors. To allow for conservative estimations of outcome reporting bias, slightly different phrasing of similar outcome variables between records and publications was allowed. Published results of primary outcomes were rated negative (supporting the null hypothesis) or positive (rejecting the null hypothesis). Prior to statistical analyses, we published our study protocol at the Open Science Framework (https://osf.io/g9mpd/?view_only=525197d231104725a15fc74253bea50b).

**TABLE 1:** Study characteristics associated with selective outcome reporting in primary outcomes

Variables	Number of trials (*N* = 151), percentage of total	Selective outcome reporting inprimary outcomes (*N* = 21 trials)	Adjusted odds ratio (AOR), (95% CI)	*p*‐value
Disorder				
Bipolar	38 (25%)	1	1 (Reference)	
MDD	51 (34%)	9	12.68 (1.40–115.2)	**0.024**
Schizophrenia	62 (41%)	11	14.53 (1.65–127.8)	**0.016**
Funding				
Industry	115 (76%)	12	1 (Reference)	
NIH	4 (3%)	0	0.00 (NA)	0.993
Other	32 (21%)	9	5.26 (1.39–19.77)	**0.014**
Centers				
Mono	29 (19%)	6	1 (Reference)	
Multi	122 (81%)	15	0.99 (0.22–4.72)	0.975
Study start year^a^ ^,^ ^b^	NA	NA	0.99 (0.84–1.18)	0.978
Anticipated enrollment^a^ ^,^ ^c^	NA	NA	0.99 (0.98–1.00)	0.657

*Note*: Significant results are shown in bold.

Abbreviations: MDD, major depressive disorder; NA, not applicable; NIH, National Institutes of Health.

^a^ Coded as a numerical variable; the odds ratio shows how much the likelihood of selective outcome reporting changes with a one‐unit increase in the numerical variable.

^b^ Study start year: Mean 2008, median 2008, range 1997–2014.

^c^ Anticipated Enrollment: Mean 602, Median 401, Range 20–18,239.

R, version 3.4.4, was used for the statistical analyses. According to previously established methods (Chan et al., 2017), multivariate logistic regression was performed with selective reporting as outcome, and funding source (categorized on ClinicalTrials.gov as industry, NIH or ‘other’, e.g., academia or unfunded), psychiatric disorder, year of study start date, participating centers, and anticipated sample size as explanatory variables, after testing for multicollinearity. Adjusted odds ratios (AOR) were calculated. Study start year and anticipated enrollment were included as numerical values, while all other variables were included as categorical values. Due to the small sample size, two‐sided Fisher exact test was used to compare the proportions of newly added positive primary outcomes with the proportions of positive results in the overall group of primary outcomes.

A post‐hoc multivariate logistic regression was performed excluding studies with an anticipated enrollment above three standard deviations from the median, to identify whether trials with large sample sizes influenced the results. This was done post‐hoc as we had not anticipated large variation in study population sizes. Following our unanticipated finding of less selective outcome reporting in industry‐funded RCTs, we used two‐sided Fisher exact test for a second and final post‐hoc test to compare changing of primary outcome measures in registry records between industry‐funded trials and non‐industry funded trials.

## 3 RESULTS

**FIGURE 1:**
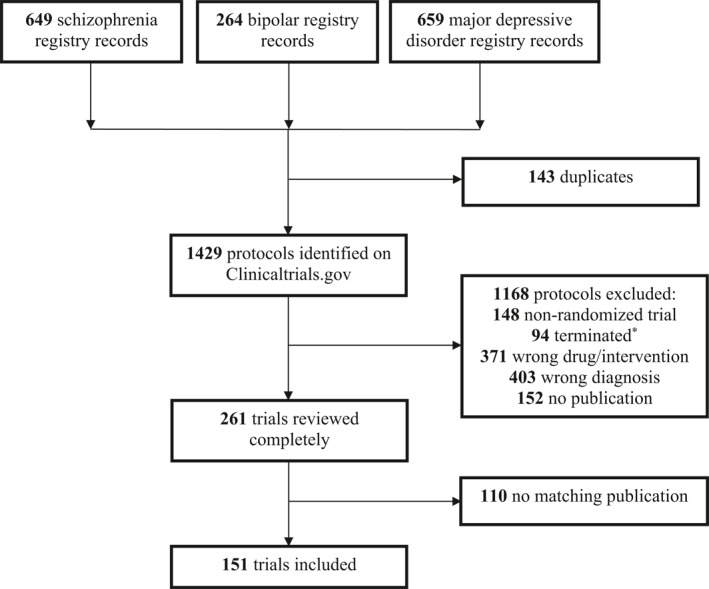
Flowchart of trial‐selection. *Terminated is defined on Clinicaltrials.gov as: “Study halted prematurely and will not resume; participants are no longer being examined or receiving intervention”

A total of 151 trials (with *N* = 94,303 participants in total) were included (Figure 1).

Of these, 21 (14%) showed irregularities between registered and published *primary* outcomes. Of the 22 added primary outcomes, 14 outcomes (64%) were ‘positive’, which was not different from the percentage of positive results in the prespecified group of primary outcomes (75%, *p* = 0.30). For *secondary* outcomes, irregularities between registration and reporting were found in 126 (83%) of included trials. 19 trials (13%) provided published outcomes entirely in line with primary and secondary outcomes as specified in Clinical Trials.gov.

Higher odds of irregularities in primary outcome measures were associated with non‐industry‐funded RCTs (AOR 5.3; *p* = 0.014) and trials investigating major depressive disorder (AOR 12.7; *p* = 0.024) or schizophrenia (AOR 14.5 and *p* = 0.016; Table 1). Study start year, number of participating centers and anticipated enrollment were not associated with selective outcome reporting (Table 1).

A post‐hoc analysis showed outcomes in registry records from industry‐funded trials were not changed more often than non‐industry funded trials (OR 0.59, *p* = 0.35), making it unlikely that updating explains high registry adherence by industry. Another post‐hoc analysis excluding studies with a large enrollment (anticipated enrollment >3 SD above the median, *N* = 3 trials) did not change the results.

## 4 DISCUSSION

We demonstrate discrepancies between trial registrations and publications in primary (14% of the RCTs) and secondary outcomes (83%) across RCTs investigating debilitating psychiatric disorders, especially in non‐industry funded RCTs. An explanation for the high rates in secondary outcomes is that they are less well conceived than the primary outcome before trial registration and therefore more prone to editing. Results for selective outcome reporting in primary and secondary outcomes are comparable to previous studies (Chan et al., 2017; Ioannidis et al., 2017; Lancee et al., 2017; Roest et al., 2015). Trials in bipolar disorder had a very low degree of selective outcome reporting relative to trials in psychotic and depressive disorders, for which we have no explanation.

Strengths of this study are the systematic assessment of both primary and secondary outcomes and adding different explanatory variables to our multivariate logistic regression. All data were separately reviewed by two investigators and discrepancies were resolved by discussions with the other co‐authors, minimizing the risk of inter‐observer variation impacting study findings.

Limitations of this study are its relatively small number of included trials, potentially limiting the statistical power needed to compute the multivariate logistic regression. Furthermore, three large psychiatric disorders were included, which may have resulted in selection bias and thereby making these results potentially not applicable to all RCTs investigating psychopharmacotherapy.

Although we included only studies on three disorders, our results point to widespread selective reporting across prevalent and debilitating psychiatric disorders. Our findings warrant further studies examining how selective reporting can be minimized, such as quality control procedures for peer reviewers and journal editors (Ioannidis et al., 2017). Increased insight into the nature of selective reporting in psychiatry and ways to tackle it will enhance the reliability of clinical trial results and optimize patient care.

## CONFLICT OF INTERESTS

None.

## Data Availability

Data are publicly available from the references listed.
